# Supplementary material for: Financing pandemic prevention, preparedness and response: lessons learned and perspectives for future
Source: Global Health. 2024 Aug 21;20:65. doi: 10.1186/s12992-024-01066-4 (PMC11337782; doi:10.1186/s12992-024-01066-4)
Supplement: Supplementary file 1 — Supplementary Material 1 [file 12992_2024_1066_MOESM1_ESM.docx]

**Financing Pandemic Prevention, Preparedness and Response: Lessons Learned and Perspectives for Future**

Nicaise Ndembi^1^, Nebiyu Dereje^1*^, Justice Nonvignon^1^, Merawi Aragaw^1^, Tajudeen Raji^1^, Mosoka Papa Fallah^1^, Mohammed Abdulaziz^1^, Benjamin Djoudalbaye^1^, Aggrey Aluso^2^, Yap Boum II^3^, Gwen Mwaba^4^, Olive Shisana^5^, Alain Ngashi Ngongo^1^, Jean Kaseya^1^

^1^Africa Centres for Disease Control and Prevention (Africa CDC), Addis Ababa, Ethiopia

^2^Pandemic Action Network, Resilience Action Network Africa, Nairobi, Kenya

^3^Institut Pasteur de Bangui, Bangui, Central African Republic

^4^ African Export-Import (Afrexim) Bank, Cairo, Egypt

^5^Deparment of Psychiatry and Mental Health, University of Cape Town, Cape Town, South Africa

*Corresponding author: Dr. Nebiyu Dereje; Email: [NebiyuDA@africacdc.org](mailto:NebiyuDA@africacdc.org)

**ABSTRACT**

**Background:** The attainment of global health security goals and universal health coverage will remain a mirage unless African health systems are adequately funded to improve resilience to public health emergencies. The COVID-19 pandemic exposed the global inequity in accessing medical countermeasures, leaving African countries far behind. As we anticipate the next pandemic, improving investments in health systems to adequately finance pandemic prevention, preparedness, and response (PPPR) promptly, ensuring equity and access to medical countermeasures, is crucial. In this article, we analyze the African and global pandemic financing initiatives and put ways forward for policymakers and the global health community to consider.

**Methods:** This article is based on a rapid literature review and desk review of various PPPR financing mechanisms in Africa and globally. Consultation of leaders and experts in the area and scrutinization of various related meeting reports and decisions have been carried out.

**Main text:** The African Union (AU) has demonstrated various innovative financing mechanisms to mitigate the impacts of public health emergencies in the continent. To improve equal access to the COVID-19 medical countermeasures, the AU launched Africa Medical Supplies Platform (AMSP) and Africa Vaccine Acquisition Trust (AVAT). These financing initiatives were instrumental in mitigating the impacts of COVID-19 and their lessons can be capitalized as we make efforts for PPPR. The COVID-19 Response Fund, subsequently converted into the African Epidemics Fund (AEF), is another innovative financing mechanism to ensure sustainable and self-reliant PPPR efforts. The global initiatives for financing PPPR include the Pandemic Emergency Financing Facility (PEF) and the Pandemic Fund. The PEF was criticized for its inadequacy in building resilient health systems, primarily because the fund ignored the prevention and preparedness items. The Pandemic Fund is also being criticized for its suboptimal emphasis on the response aspect of the pandemic and non-inclusive governance structure.

**Conclusions:** To ensure optimal financing for PPPR, we call upon the global health community and decision-makers to focus on the harmonization of financing efforts for PPPR, make regional financing mechanisms central to global PPPR financing efforts, and ensure the inclusivity of international finance governance systems.

**Keywords:** Pandemic; Fund; Financing; Global Health Security; PPPR

**BACKGROUND**

The health systems in Africa, already limited in capability and capacity, face the daunting challenge of addressing emerging and reemerging public health emergencies.^1^ This challenge is set against a backdrop of economic vulnerabilities, including significant debts and populations with generally low socioeconomic status and health literacy. Infectious diseases still have a severe impact on the African continent, accounting for over 227 million years of healthy life lost every year and producing an annual productivity loss of over $800 billion.^1^ Compounding this situation are the disproportionate impacts of climate change on public health and the ongoing wars and conflicts in various regions of the continent.^2^ Such circumstances severely impede Africa’s ability to effectively engage in pandemic prevention, preparedness, and response (PPPR).

Building strong, resilient health systems in Africa that can adequately handle public health emergencies is crucial. However, the severe underfunding of health systems, as evidenced by the low domestic investments of African governments, contrary to the commitments of the Abuja declaration, specifically on spending 15% of the budget on health, could further worsen the already weak health systems.^3^ The attainment of global health security (GHS) goals and universal health coverage (UHC) will remain a mirage unless African health systems are adequately and efficiently funded to improve resilience to emerging and reemerging public health emergencies. GHS agenda mainly targets infectious disease prevention and control while the UHC prioritizes universal, timely, and quality access to essential healthcare services for everyone.^4,5^ However, both initiatives have impacts on strengthening one another. A strong health system built by the UHC can significantly contribute to the success of the GHS agenda and vice-versa.^4,6,7^ To ensure this, various scholars indicated the need for integration (building capacity for GHS within the comprehensive UHC framework), investment (unified financing to strengthen the overall health system), building a resilient health system, and addressing the inequity gaps.^4–9^ This is critical in Africa where the infectious disease burden is high and resources are limited.

As we anticipate the next pandemic, improving investments in health systems and building a solid buffer to adequately finance PPPR promptly, ensuring equity and access to medical counter-measures is crucial, and mechanisms must be placed in the ongoing Pandemic Agreement negotiations.^10,11^ The pandemic financing system is a fundamental element to ensure the realization of the envisioned safe and equitable world for everyone by the Pandemic Agreement.^11^ In this article, we analyzed the African and global pandemic financing initiatives and put ways forward for the consideration of policymakers and the global health community.

**Methods**

This article is based on a rapid literature review and desk review of financial mechanisms implemented to address the pandemic prevention preparedness and response in Africa and globally. We used Google, Google Scholar, PubMed Central, and Web of Sciences to search for relevant documents, reports, and published journal articles stating PPPR financial mechanisms. We also made consultations with prominent experts and leaders of various institutions such as the Africa Union, Africa CDC, World Bank, Afriexim Bank, and Pandemic Action Network to get their perspectives and insights into the financing mechanisms. Moreover, we searched various institutions’ websites that are relevant to our topic of inquiry such as the World Bank website for PPPR financial initiatives including the Pandemic Fund, the Africa Union website for the COVID-19 Response Fund, and the Epidemic Intelligence Fund. We scrutinized various meeting reports and decisions to support our analysis of the financial mechanisms.

**MAIN TEXT**

**African Union Initiatives to Finance the PPPR**

With the challenges presented by public health emergencies such as the West African Ebola outbreak and the COVID-19 pandemic, the African continent has learned important lessons that need to be translated into policy and action as part of PPPR. The West African Ebola outbreak exposed African health systems' fragility and reliance on international expertise and support to respond to the outbreak – underscoring the overwhelming urgent need for building local capacity to effectively mobilize domestic resources (health workforce, finance, and leadership).^12^

The recent COVID-19 response unveiled serious global inequities regarding access to medical countermeasures (vaccines, diagnostics, and therapeutics), and Africa was left far behind. The global financing systems utterly failed to fulfill the demands of the countries as evidenced by an alarmingly more than 100-day gap between the first COVID-19 vaccination in low-income countries (LICs) and high-income countries (HICs)^13^, and an average daily testing capacity of 6.07 tests per 1000 people in HICs as compared to 0.08 tests per 1000 people in LICs.^14^ The African Union (AU) initially dealt with the COVAX (COVID-19 Vaccine Global Access) - Access to COVID-19 Tools Accelerator (ACT-A) to address the vaccine access gap. However, the COVAX facility is said to have been severely delayed in ensuring that vaccines are optionally supplied to Africa and other LMICs.^15^ ACT-A was an unprecedented global coordination mechanism co-chaired by South Africa and Norway, which raised 24 billion dollars, distributed over vaccines, diagnostics, therapeutics and other essential medical products. Notwithstanding its phenomenal success, there were serious and life-costing pitfalls: it took too long to raise the financing, vaccine deployment was delayed by issues of export bans and other geopolitical tensions, the diagnostics and therapeutics pillars did not meet their targets, and the health systems connector pillar did not operationalize adequately and failed to meet its mandate, compromising critical last mile capabilities (Table 1). As a result, the ACT-A fell short of delivering equity, as evidenced by the fact that today, the world average for vaccination is 67%, while low-income countries only average 27%.^16^ According to the ACT-A external evaluation, a similar platform for future pandemics should have, inter alia, better coordination on R&D, that there should be available contingent funding on Day Zero of the next pandemic, that there should be a ‘strong representation of regional actors’ in the governance structure, and a stronger emphasis on technology transfer.^17^

During the pandemic, the African Union acted swiftly to mitigate inequities, including by creating a pooled vaccine and medical supplies procurement platforms, Africa Vaccine Acquisition Trust (AVAT) and Africa Medical Supplies Platform (AMSP) – successfully delivering medical countermeasures to the AU Member States (Table 1).

The AMSP is a digital platform that unlocks immediate access to an African and global base of vetted manufacturers and enables AU Member States to purchase certified medical equipment and clinical management devices with increased cost-effectiveness and transparency. The platform is a unique interface enabling volume aggregation, payment facilitation, and logistics and transportation to ensure equitable and efficient access to critical supplies for African governments. The AVAT is a special-purpose vehicle created to facilitate the pooled procurement mechanism and act as the interface between AU member states and vaccine manufacturers. The procurement of COVID-19 vaccines through AVAT was supported by a $2 billion financial guarantee issued by the African Export-Import Bank (Afreximbank). AVAT negotiated and executed a vaccine supply contract with Johnson & Johnson (J&J) for 220 million vaccine doses on a committed basis (at a cost of $7.5 per dose for a total of USD 1.65 billion) with an option to trigger an additional 180 million vaccine doses subject to demand from AU Member States. With the backing of a payment guarantee from Afreximbank, AVAT was able to meet all its financial obligations to the vaccine manufacturer J&J. All J&J invoices for supplies made by J&J were settled by AVAT on behalf of AU Member States. As of 8^th^ May 2024, 158.3 million doses have been shipped to the participating countries (42 African countries plus 6 Caribbean Community and Common Market (CARICOM) countries). A further quantity of 30.3 million doses has been accepted by the countries and in the process of being prepared for shipment. Strong strategic partnerships and collaborations with institutions and organizations like the World Bank, Mastercard Foundation, Africa CDC, UNICEF, and MTN amongst others who worked with AVAT’s pooled procurement mechanism initiative a success.

This guarantee provided payment assurance to the vaccine manufacturers.^18^ However, financial constraints and reduced vaccine uptake in Member States after the end of the declaration of COVID-19 as a Public Health Emergency of International Concern (PHEIC) challenged the initiatives.

The COVID-19 Response Fund was another significant AU initiative, that demonstrated the role of public-private partnership (PPP) in strengthening domestic capacity to mitigate the impact of the pandemic. The partnership aimed to raise an initial $150 million for immediate needs to prevent the spread and up to $400 million to support sustainable medical response to the COVID-19 pandemic by pooling the resources required for the procurement of medical supplies and commodities, supporting the deployment of rapid responders across the continent as well as providing socio-economic support to the most vulnerable populations in Africa.

Capitalizing on lessons learned from this initiative, heads of state of the AU member states converted the fund into the African Epidemics Fund (AEF), which was envisioned to support the African continent's sustainable and self-reliant PPPR efforts. The AEF is set to be mobilized from various sources, including contributions from the Member States, private sectors, philanthropies, and international stakeholders. Unlike other ear-marked and vertical funding, this fund received through the AEF shall be used flexibly to accommodate the continent's evolving needs, thereby continually strengthening Member States' capability towards effective and timely PPPR efforts.

The other AU initiative, the African Risk Capacity (ARC), aims to support AU Member States in improving their capacities to better plan, prepare, and respond to weather-induced events and disease outbreaks.^19^ ARC is the parametric sovereign insurance policy that is contributed by Member States so that the insurance supports outbreak response efforts of Member States by providing predictable funding at the early response phase of the outbreak or epidemic. To date, 39 AU Member States have ratified the APC treaty and signed membership, and 62 policies have been signed by the Member States for cumulative insurance coverage of US $720 million to protect 72 million vulnerable populations in participating countries.^20^ Strengthening these domestic financing mechanisms is essential for creating a sense of ownership in the countries and ensuring sustainable funds for PPPR.

**Table 1: Summary of Africa Union Initiatives to Finance PPPR**

| **Financing mechanism (initiative)** | **Objectives** | **Date of set up** | **Status** | **Levels of performance** | **Weakness** |
| --- | --- | --- | --- | --- | --- |
| COVAX Access to COVID-19 Tools Accelerator (ACT-A) | To accelerate development, production, and equitable access to COVID-19 tests, treatments, and vaccines | April 2020 | Ended | Able to collect $24 billion; procured and distributed >2 billion doses of COVID-19 vaccines; distributed diagnostics therapeutics and PPEs, supported 70+ countries in expanding laboratory infrastructure and ramping up testing; formed COVID-19 Oxygen Emergency Taskforce; immediate contribution of up to US$ 20 million to kick off the emergency response | Severely delayed providing vaccines to Africa and LMICs, it took too long to raise the financing, vaccine deployment was delayed by issues of export bans and other geopolitical tensions, the diagnostics and therapeutics pillars did not meet their targets, and the health systems connector pillar did not operationalize adequately and failed to meet its mandate, compromising critical last mile capabilities. |
| Africa Medical Supplies Platform (AMSP) | To enable AU Member States to purchase certified medical equipment and clinical management devices with increased cost-effectiveness and transparency | April 2020 | Active | Able to create a platform to liaise the procurement systems of the countermeasures (PPEs, Testing kits, vaccines, and therapeutics).  Was also able to support the logistics and deliveries of the AVAT COVID-19 vaccines.  Provided the required coordination platform to AU Member States on behalf of AVAT. | Funding limitation is needed to increase the supply capacity of their customer base. |
| Africa Vaccine Acquisition Trust (AVAT) | To facilitate the pooled procurement mechanism and act as the interface between AU member states and vaccine manufacturers | August 2020 | Active | Negotiated and executed 220 million Johnson & Johnson (J&J) vaccine supply, with a total cost of $1.65 billion and as of 8^th^ May 2024, 158.3 million doses have been shipped to the participating countries. | Reduced vaccines uptake from the member states, especially since COVID-19 was declared to be no longer a Public Health Emergency of International Concern (PHEIC).  There are challenges in getting outstanding payments from some of the self-funded member countries. |
| COVID-19 Response Fund | To raise finances to support immediate needs to prevent the spread and sustain the medical response to the COVID-19 pandemic | Jan 2020 | Transitioned into African Epidemic Fund | Collected $146 million from Member states’ and partners’ contributions and disbursed $74 million as part of the COVID-19 response | Lack of flexibility to use the fund to address other public health emergencies |
| African Epidemics Fund (AEF) | To support the African continent's sustainable and self-reliant PPPR efforts | Feb 2022 | Active | Launched and the Fund's financial resources shall consist of: (a) a startup amount from the transfer of the outstanding balance of the COVID-19 Response fund; (b) Voluntary contributions of the AU Member States, individuals, and any entity or organ from AU; (c) contribution from International Partners(( (d) contributions from non-African partners; (e) income generated from prudent investment of the funds; (f) Private sector fund mobilization; and (g) Innovative Financing (crowdfunding, administrative charges, e.t.c.; and (h) any unused funds upon completion of an action. | As it is a new initiative, reports on its limitations have not been assessed. |
| African Risk Capacity (APC) | To support Member States improve their capacities to better plan, prepare and respond to weather-induced events and disease outbreaks | Nov 2012 | Active | To date, 39 AU Member States have ratified the APC treaty and signed membership, and 62 policies have been signed by the Member States for cumulative insurance coverage of US $720 million for the protection of 72 million vulnerable populations in participating countries. | Delayed ratification and signing of insurance policy in some of the AU Member States |

**The Global Financing Initiatives for PPPR**

As diseases do not respect international borders, it is a must for global communities to make concerted and collaborative efforts to ensure global health security. The COVID-19 pandemic has practically demonstrated that ‘no one is safe until everyone is safe’. Under this premise, several global initiatives, including the Pandemic Fund, have been established to finance the PPPR. The Pandemic Fund seeks to channel critical investments to strengthen PPPR capacities at national, regional, and global levels, particularly in low- and middle-income countries – envisioning a resilient world. The Pandemic Fund has been contributed by donor countries, co-investors, and foundations, and hosted by the World Bank as a trustee.^21^

The World Bank has been indeed engaged in various initiatives to support pandemic response, such as the response to the 2014–16 Ebola outbreak in West Africa and the establishment of the Pandemic Emergency Financing Facility (PEF) to fund outbreak responses (started in 2016 but now ceased operation in April 2021). These initiatives of the World Bank were instrumental in providing the required support for the response to the outbreaks, although they were criticized for their inadequacy in building resilient health systems that can go beyond the emergency period. Particularly, the prevention and preparedness items were ignored in these initiatives.^22^The PEF comes to its end following the recommendation of the International Working Group of the World Bank, underscoring the need to strengthen and scale up investments in global health security as an urgent priority.^23^

To address the gaps in financing PPPR in countries, the World Bank established an International Working Group in Financing Pandemic Preparedness in 2016, with a mandate to propose how national governments and partners can ensure optimal and sustainable financing for actions to strengthen PPPR.^23^ In May 2017, the World Bank International Working Group in Financing Pandemic Preparedness proposed strategies to finance preparedness and response capacities for pandemics and other health emergencies, particularly underscoring the need for national governments to increase domestic finances, development partners to capitalize on the existing bi-lateral and multilateral collaborations to finance preparedness and response, and the World Bank and International Monitory Fund (IMF) to place strategies to incentivize countries to invest in preparedness.^23^ In 2019, the Center for Strategic and International Studies (CSIS) recommended new multilateral PPR financing, underscoring the need for the U.S. government to establish a Pandemic Preparedness Challenge at the World Bank to incentivize countries to invest in their preparedness. These initiatives and recommendations were critical in shaping the global financing architecture for PPPR.^24^ Following the declaration of COVID-19 as a Public Health Emergency of International Concern (PHEIC) in 2020 by the World Health Organization (WHO), global leaders realized the vitality of having a strong and sustainable financing mechanism for PPPR to ensure global health security. In January 2021, G20 nations established a High-Level Independent Panel (HLIP) to recommend financing the global commons for PPPR. The HLIP strongly recommended countries make substantial investments, more than ever committed, to avoid the next pandemic – further pronouncing the critical need for increased domestic financing (up to an additional 1% of the GDP), increased additional international financing of up to $15 billion per year, and addressing the critical gap in global health governance architecture by integrating key actors, both from the global health and financing systems.^25,26^

Drawing from these evolving outbreak response lessons and consultations, the Pandemic Fund has come to its realization (Figure 1). The Pandemic Fund has adopted a multilateral common goods financing approach to address persistent global health challenges, as demonstrated by the establishment of the Global Fund to combat HIV/AIDS, Tuberculosis and Malaria, and GAVI, the Vaccine Alliance. ^27^

Figure 1: Prominent global milestones in the journey to support PPPR

The global PPPR requires an estimated $31.1 billion annual investment. Considering the existing and potential international and domestic financing for PPPR, it is estimated that at least an additional $10.5 billion per year in international financing will be needed to fund a fit-for-purpose PPPR, with a substantial gap in the LICs.^26^ However, only $1.9 billion was pledged for the Pandemic Fund by donors and partners as of May 2023, and only $300 million was disbursed in the first round of pandemic funding – which was too far from the demand from LMICs that exceeded $7 billion.^28^ This demand for funds requested from LMICs, with aims to strengthen their disease surveillance and early warning systems, laboratory systems, and health workforce development, implies their commitment and intense desire to prepare for the next pandemic. However, only five African countries were funded in the first round of the fund, and there was no consideration of regional entities from Africa. Moreover, the Pandemic Fund has been criticized for its suboptimal emphasis on the response aspect of public health emergencies.^29^ More importantly, the governance structure of the Pandemic Fund needs to be all-inclusive, whether in high-income or low-income countries, rich or resource-constrained settings.^25–27,29^ Surge financing from Day Zero has been proposed by the G7 countries, to ensure the immediate release of pre-arranged finance for countermeasures at the onset of the next pandemic.^30^ Notably, 60–75% of the delay in COVID-19 vaccine access to LMICs was attributable to their signing procurement agreements later than high-income countries, which placed them further behind in the delivery line.^17^ While supporting the cruciality of availing pre-arranged funds for immediate release, we also advocate that the initiative must be designed to ensure timely and equitable access to countermeasures to all countries.

Other remarkable initiatives such as the Global Health Initiatives (Global Fund, PEPFAR, World Bank and GAVI), established in 2002 to raise and disburse funds to address infectious diseases, immunization and strengthen the health systems in LMICs, have made several strides to support global health security and universal health coverage. However, due to the changes in global health needs, financing and governance, reshaping global health architecture is critical to building a more robust and resilient health system that can cope with emerging public health threats while enhancing everyone's access to essential healthcare services.^31^

Under these premises, delegates from multi-sectoral organizations (funders, governments, global health organizations, civil society, and the research and learning community) had 14 months of engagements to develop strategic shifts on GHIs to address the challenges of UHC and global health security sustainably. These engagements resulted in the “Lusaka Agenda” which proposes five strategic shifts – make a more substantial contribution to primary health care (PHC) by effectively strengthening systems for health; play a catalytic role towards sustainable, domestically-financed health services and public health functions; strengthen joint approaches for achieving equity in health outcomes; achieve strategic and operational coherence; coordinate approaches to products, research and development (R&D), and regional manufacturing to address market and policy failures in global health. To realize this strategic shift, a more inclusive and transparent governance system, monitoring the impacts of the initiatives, and working closely with government systems are required.^31^

**A Call to Action**

As we are in the season where pandemics emerge and reemerge more frequently and spread more quickly, it is imperative to place mechanisms that ensure effective and sustainable financing for PPPR. As such, we call upon the global health community and decision-makers to focus on the harmonization of financing efforts for PPPR. The proliferation of (multiple) financing mechanisms for pandemics does not focus efforts but diverts attention and resources. Moreover, fragmented multiple financing mechanisms make financial and programmatic monitoring complex and challenging.^32^ Thus, it is critical to make financial investments harmonized in a way that contributes to building comprehensive and resilient health systems to address current and future public health emergencies.

There is an urgent need to make regional financing mechanisms central to global PPPR financing efforts. The inequity we experienced with the COVID-19 response can only be addressed with regional financing that can optimally support PPPR initiatives such as geographically diversified and sustainable production of countermeasures. So, for equity in access and flexibility, AEF and other regional efforts need to be the focus and perhaps entrenched in global PPPR financing discussions. AEF can serve as a financing entity to support African Union member states in their efforts for pandemic PPR. The global entities, funders, partners, and philanthropies can provide direct financial support to this initiative. Notably, countries have easier access and a sense of ownership to regional mechanisms than global ones.

It is a must for the local and global financing mechanisms to adequately and proportionally align to support the public health threats, and the international finance governance systems ensure the inclusivity of all the key actors, irrespective of their development status to ensure global health security. The engagement of private sectors and philanthropies in the financing efforts must be considered.

We need to fortify our defenses against future pandemics by investing in comprehensive Pandemic Prevention, Preparedness, and Response strategies. Financial support is crucial to developing robust health systems, advancing research for rapid diagnostics, vaccines, and treatments, and establishing resilient supply chains for critical medical supplies. We must build a safer world by being part of the shield protecting humanity from the next health crisis.

**CONCLUSIONS**

As we are in the season with enormous emerging and reemerging public health threats, it is imperative to place mechanisms that ensure equity, and effective and sustainable financing for PPPR. Lessons learned from the continental and global financing initiatives, particularly during the COVID-19 pandemic, must be translated as we prepare for the next pandemic. Therefore, we call upon the global health community and decision-makers to focus on the harmonization of financing efforts for PPPR, make regional financing mechanisms central to global PPPR financing efforts, and ensure the inclusivity of international finance governance systems. This can be realized through provisions that can be included in the Pandemic Agreement.

**List of abbreviations**

AEF: Africa Epidemic Fund

AMSP: Africa Medical Supplies Platform

AVAT: Africa Vaccine Acquisition Trust

AU: African Union

COVID-19: Coronavirus Disease

HICs: High-income Countries

LICs: Low-income Countries

PEF: Pandemic Emergency Financing Facility

PPPR: Pandemic Prevention, Preparedness and Response

WHO: World Health Organization

**Declarations**

**Ethics Approval and Consent to Participate**: Not applicable

**Consent for Publication**: Not applicable

**Availability of Data and Materials**: Not applicable.

**Competing Interests:** The authors declare no competing interests.

**Funding:** The write-up of this manuscript is supported by Africa Centres for Disease Control and Prevention (Africa CDC) through funding from the Mastercard Foundation.

**Author’s Contributions:** NN and ND conceptualized the study and drafted the original manuscript, JN, MA, TR, MPF, BD, AL, YB, GW, OS, ANN, and JK critically reviewed the manuscript, and all the authors have reviewed and approved the final version of the manuscript.

**Acknowledgement:** Not applicable.

**REFERENCES**

1. Nkengasong JN, Tessema SK. Africa needs a new public health order to tackle infectious disease threats. *Cell*. 2020;183(2):296-300.

2. Kaseya J, Dereje N, Raji T, Ngongo AN, Fallah MP, Ndembi N. Public health emergencies in war and armed conflicts in Africa: What is expected from the global health community? *BMJ Glob Health*. 2024;9(3):e015371.

3. African Union. *Abuja Declaration on HIV/AIDS, Tuberculosis and Other Related Infectious Diseases*.; 2001. chrome-extension://efaidnbmnnnibpcajpcglclefindmkaj/https://au.int/sites/default/files/pages/32894-file-2001-abuja-declaration.pdf

4. Wenham C, Katz R, Birungi C, et al. Global health security and universal health coverage: from a marriage of convenience to a strategic, effective partnership. *BMJ Glob Health*. 2019;4(1):e001145.

5. Debie A, Khatri RB, Assefa Y. Successes and challenges of health systems governance towards universal health coverage and global health security: a narrative review and synthesis of the literature. *Health Res Policy Syst*. 2022;20(1):50.

6. Erondu NA, Martin J, Marten R, Ooms G, Yates R, Heymann DL. Building the case for embedding global health security into universal health coverage: a proposal for a unified health system that includes public health. *The Lancet*. 2018;392(10156):1482-1486.

7. Ooms G, Beiersmann C, Flores W, et al. Synergies and tensions between universal health coverage and global health security: why we need a second ‘Maximizing Positive Synergies’ initiative. *BMJ Glob Health*. 2017;2(1):e000217. doi:10.1136/bmjgh-2016-000217

8. Assefa Y, Hill PS, Gilks CF, et al. Global health security and universal health coverage: Understanding convergences and divergences for a synergistic response. *PLoS One*. 2021;15(12):e0244555. https://doi.org/10.1371/journal.pone.0244555

9. Lal A, Erondu NA, Heymann DL, Gitahi G, Yates R. Fragmented health systems in COVID-19: rectifying the misalignment between global health security and universal health coverage. *The Lancet*. 2021;397(10268):61-67.

10. World Health Organization. Governments agree to continue their steady progress on proposed pandemic agreement ahead of the World Health Assembly. Published 2024. https://www.who.int/news/item/10-05-2024-governments-agree-to-continue-their-steady-progress-on-proposed-pandemic-agreement-ahead-of-the-world-health-assembly

11. Ndembi N, Dereje N, Rahman FA, et al. The pandemic agreement: Achieving an African win for health security inequity. *J Public Health Afr*. 2024;15(1):618.

12. Fallah MP. To beat Ebola in Uganda, fund what worked in Liberia. *Nature*. Published online 2022:427.

13. Duroseau B, Kipshidze N, Limaye RJ. The impact of delayed access to COVID-19 vaccines in low-and lower-middle-income countries. *Front Public Health*. 2023;10:1087138.

14. Narayanasamy S, Okware B, Muttamba W, et al. Global inequity of COVID-19 diagnostics: challenges and opportunities. *J Epidemiol Community Health*. 2022;76(12):972-975.

15. Pushkaran A, Chattu VK, Narayanan P. A critical analysis of COVAX alliance and corresponding global health governance and policy issues: a scoping review. *BMJ Glob Health*. 2023;8(10):e012168.

16. World Health Organization. WHO Coronavirus (COVID-19) Dashboard. https://covid19.who.int/.

17. Agarwal R, Reed T. *Finance Vaccine Equity: Funding for Day-Zero of the next Pandemic*. International Monetary Fund; 2022.

18. Fallah MP, Raji T, Ngongo AN, et al. The role of Africa Centres for Disease Control and Prevention during response to COVID-19 pandemic in Africa: lessons learnt for future pandemics preparedness, prevention, and response. *BMJ Glob Health*. 2024;9(2):e014872. doi:10.1136/bmjgh-2023-014872

19. African Risk Capacity. African Risk Capacity: Sovereign Disaster Risk Solutions. Published 2024. https://www.arc.int/about

20. African Risk Capacity. Impact. Published 2024. https://www.arc.int/impact

21. The World Bank. The Pandemic Fund. https://fiftrustee.worldbank.org/en/about/unit/dfi/fiftrustee/fund-detail/pppr

22. Brim B, Wenham C. Pandemic Emergency Financing Facility: struggling to deliver on its innovative promise. *BMJ*. 2019;367.

23. The World Bank. *From Panic and Neglect to Investing in Health Security: Financing Pandemic Preparedness at a National Level*. https://www.worldbank.org/en/topic/pandemics/publication/from-panic-neglect-to-investing-in-health-security-financing-pandemic-preparedness-at-a-national-level

24. Ayotte K, Gerberding J, Morrison JS. *Ending the Cycle of Crisis and Complacency in US Global Health Security: A Report of the CSIS Commission on Strengthening America’s Health Security*. Center for Strategic & International Studies; 2020.

25. Micah AE, Bhangdia K, Cogswell IE, et al. Global investments in pandemic preparedness and COVID-19: development assistance and domestic spending on health between 1990 and 2026. *Lancet Glob Health*. 2023;11(3):e385-e413.

26. Panel HLI. A global deal for our pandemic age. *Report of the G20 High Level Independent Panel on Financing the Global Commons for Pandemic Preparedness and Response, https://pandemic-financing org/report/foreword*. Published online 2021.

27. Reid-Henry S, Lidén J, Benn C, Saminarsih D, Herlinda O, Venegas MFB. A new paradigm is needed for financing the pandemic fund. *The Lancet*. 2022;400(10349):345-346.

28. The World Bank. Demand for funding from Pandemic Fund exceeds expectations with requests totaling over $7 billion. https://www.worldbank.org/en/programs/financial-intermediary-fund-for-pandemic-prevention-preparedness-and-response-ppr-fif/brief/demand-for-funding-from-pandemic-fund-exceeds-expectations-with-requests-totaling-over-7-billion

29. Boyce MR, Sorrell EM, Standley CJ. An early analysis of the World Bank’s Pandemic Fund: a new fund for pandemic prevention, preparedness and response. *BMJ Glob Health*. 2023;8(1):e011172. doi:10.1136/bmjgh-2022-011172

30. U.S. DEPARTMENT OF THE TREASURY. G7 Finance Ministers and Central Bank Governors’ Communiqué. Published 2024. https://home.treasury.gov/news/press-releases/jy2371

31. Future of Global Health Initiatives. *The Lusaka Agenda: Conclusions of the Future of Global Health Initiatives Process*.; 2024. https://futureofghis.org/final-outputs/lusaka-agenda/

32. Brown GW, Rhodes N, Tacheva B, Loewenson R, Shahid M, Poitier F. Challenges in international health financing and implications for the new pandemic fund. *Global Health*. 2023;19(1):97.
